# Meta-analysis of molecular response of kidney to ischemia reperfusion injury for the identification of new candidate genes

**DOI:** 10.1186/1471-2369-14-231

**Published:** 2013-10-24

**Authors:** Dmitry N Grigoryev, Dilyara I Cheranova, Daniel P Heruth, Peixin Huang, Li Q Zhang, Hamid Rabb, Shui Q Ye

**Affiliations:** 1 Division of Experimental and Translational Genetics, Department of Pediatrics, Children’s Mercy Hospitals and Clinics, University of Missouri School of Medicine, Kansas City, MO, USA; 2 Department of Biomedical and Health Informatics, University of Missouri School of Medicine, Kansas City, MO, USA; 3 Omics Analysis Unit, Core of Genetic Research, Division of Experimental and Translational Genetics, Department of Pediatrics, Children’s Mercy Hospitals and Clinics, University of Missouri-Kansas City School of Medicine, Kansas City, MO 64108, USA; 4 Division of Nephrology, Department of Medicine, Johns Hopkins University, Baltimore, MD, USA

**Keywords:** Kidney, Ischemia reperfusion injury, Bioinformatics, Meta-analysis

## Abstract

**Background:**

Accumulated to-date microarray data on ischemia reperfusion injury (IRI) of kidney represent a powerful source for identifying new targets and mechanisms of kidney IRI. In this study, we conducted a meta-analysis of gene expression profiles of kidney IRI in human, pig, rat, and mouse models, using a new scoring method to correct for the bias of overrepresented species. The gene expression profiles were obtained from the public repositories for 24 different models. After filtering against inclusion criteria 21 experimental settings were selected for meta-analysis and were represented by 11 rat models, 6 mouse models, and 2 models each for pig and human, with a total of 150 samples. Meta-analysis was conducted using expression-based genome-wide association study (eGWAS). The eGWAS results were corrected for a rodent species bias using a new weighted scoring algorithm, which favors genes with unidirectional change in expression in all tested species.

**Results:**

Our meta-analysis corrected for a species bias, identified 46 upregulated and 1 downregulated genes, of which 26 (55%) were known to be associated with kidney IRI or kidney transplantation, including *LCN2*, *CCL2*, *CXCL1*, *HMOX1*, *ICAM1*, *ANXA1*, and *TIMP1*, which justified our approach. Pathway analysis of our candidates identified “*Acute renal failure panel*” as the most implicated pathway, which further validates our new method. Among new IRI candidates were 10 novel (<5 published reports related to kidney IRI) and 11 new candidates (0 reports related to kidney IRI) including the most prominent candidates *ANXA2*, *CLDN4*, and *TYROBP*. The cross-species expression pattern of these genes allowed us to generate three workable hypotheses of kidney IRI, one of which was confirmed by an additional study.

**Conclusions:**

Our first in the field kidney IRI meta-analysis of 150 microarray samples, corrected for a species bias, identified 10 novel and 11 new candidate genes. Moreover, our new meta-analysis correction method improved gene candidate selection by identifying genes that are model and species independent, as a result, function of these genes can be directly extrapolated to the disease state in human and facilitate translation of potential diagnostic or therapeutic properties of these candidates to the bedside.

## Background

There is accumulating evidence for the direct correlation between duration of donor organ ischemia and rejection rates [1,2]. Therefore, the development of protective therapies for ischemia reperfusion injury (IRI) of transplanted organs is of great importance. However, the mechanisms by which IRI predisposes to allograft injury are poorly understood. To capture this complex process, global gene expression profiling has been intensively utilized and its results made available to public. The accumulated data represent a remarkable substrate for the analysis of global molecular changes invoked by kidney IRI in different experimental settings. However, most of the kidney IRI data is generated using rodent experimental models. Therefore, we hypothesized that correction of meta-analysis of IRI-implicated genes for a species bias will not only improve new gene candidate selection, but will identify genes that are model and species independent. Therefore, the new diagnostic or therapeutic properties discovered for these genes will be easier translated to a clinical use.

## Results and discussion

The 21 experimental settings used for our meta-analysis comprised 150 samples (65 shams and 85 IRIs). The combined data set had 12 post-ischemia time-points represented by five different platforms (ABI, Affymetrix, Agilent, Amersham, and Illumina) and four species (human, pig, rat, and mouse) and was stratified by the recovery time after ischemia (Table 1).

**Table 1:** First tier IRI gene candidates

**Gene**	**Symbol**	***χ***^***2 ***^**P value**	***W *** **score**	**PubMatrix search terms**		
				**Kidney injury**	**Kidney ischemia reperfusion injury**	**Kidney transplantation**
Lipocalin 2	LCN2	2.36E-11	33.3	314	38	57
TYRO tyrosine kinase binding protein	TYROBP	5.54E-08	22.3	2	1	1
Annexin A1	ANXA1	4.52E-14	27	3	3	1
Chemokine (C-C motif) ligand 2	CCL2	3.97E-11	36	313	56	96
Heme oxygenase (decycling) 1	HMOX1	3.97E-11	27	291	100	110
Clusterin	CLU	3.97E-11	25.5	27	1	11
Early growth response 2	EGR2	1.43E-10	19	0	0	0
Cyclin-dependent kinase inhibitor 1A	CDKN1A	2.34E-10	26	83	16	28
Activating transcription factor 3	ATF3	1.38E-09	29	5	5	1
Chemokine (C-X-C motif) ligand 1	CXCL1	1.38E-09	25	32	18	11
ADAM metallopeptidase with TS1	ADAMTS1	1.75E-09	29	2	1	1
RAS, dexamethasone-induced 1	RASD1	5.33E-09	18	1	1	1
TIMP metallopeptidase inhibitor 1	TIMP1	8.11E-09	31	162	57	163
TNF receptor superfamily, member 12A	TNFRSF12A	9.88E-09	32.5	9	1	2
Jun proto-oncogene	JUN	5.52E-08	23	134	37	36
Filamin A, alpha	FLNA	5.52E-08	19	0	0	0
Intercellular adhesion molecule 1	ICAM1	5.52E-08	18	352	131	244
Secreted phosphoprotein 1	SPP1	5.54E-08	24	95	7	22
Lectin, galactoside-binding, soluble, 3	LGALS3	5.54E-08	21	11	4	3
Baculoviral IAP repeat-containing 3	BIRC3	1.66E-06	14	1	0	1

**Figure 1:**
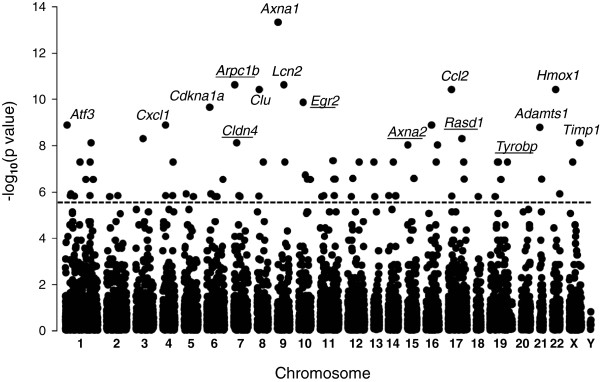
**eGWAS for kidney IRI using a *χ***^**2 **^**analysis.** Plot of (-log_10_P value) (y axis) by chromosomal location (x axis). P values for each gene were calculated for 21 microarray experiments with 65 sham-operated or pre-transplant control microarray samples and 85 IRI or post-transplant microarray samples as the likelihood of finding repeated differential expression compared with expected using ***χ***^*2*^ analysis. Out of tested 17,575 genes 75 genes demonstrated a significant differential expression. The dotted line indicates the Bonferroni threshold (P = 2.84 × 10^-6^). The genes symbols indicate genes that are most significantly associated with kidney IRI, where underscored symbols indicate new candidate genes.

**Figure 2:**
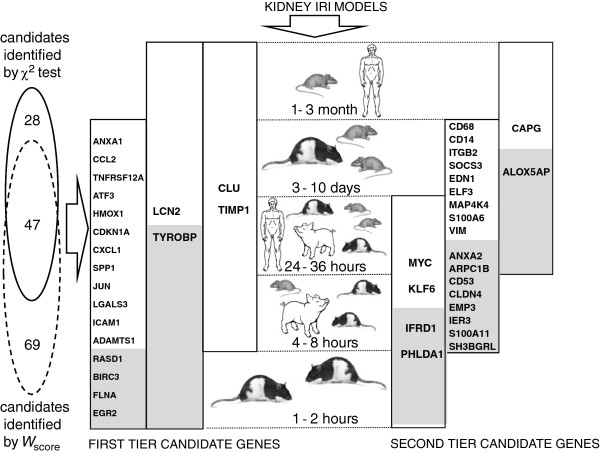
**Analytical arrangement of available genomics studies of IRI in kidney and resulting lists of major IRI-associated candidate genes.** Seventeen global transcriptional profiles were grouped into five clusters according to post-ischemia time. The species composition of each cluster is represented by a corresponding mammal. Vertical bars represent extend of significant expression of a particular gene throughout the five time-points. 46 upregulated and 1 downregulated candidate genes were selected by cross-referencing gene lists generated with χ^*2*^ test and *W*-scoring procedures (Venn diagram on the left). First tier candidate genes are depicted on the left and were significantly affected by IRI in at least four consecutive time-points. Each bar is filled with the corresponding gene symbol; where the open part of a bar is populated with the well known kidney IRI genes and grey area of the bar cover novel and new kidney IRI genes. The second tier candidate genes are listed on the right with the same color coding. The full names of depicted genes can be found in Table 1 (first tier) and Additional file 3. * The 4 genes from the third tier candidates (JUNB, ↓UPB1, CXCL10, CEBPD) are not included into the figure.

The cross-referencing microarray platforms resulted in universal 17,575 genes, which were analyzed by eGWAS. The eGWAS ranked genes by the likelihood that repeated differential expression for a given gene was due to chance, then controlled for multiple-hypothesis testing using Bonferroni threshold (P < 2.84 × 10^-6^). The eGWAS identified 75 candidate genes (Figure 1, Additional file 1). To correct this result for species and platform bias, these 75 genes were cross-referenced against 116 candidates identified by the weighted scoring (*W*-score) algorithm (Figure 2, Additional file 2). The resulting 47 genes were divided into three tiered candidate lists (Figure 2, Additional file 3). First tier contains 20 genes, which responded to IRI in all time points, or in 4 consequent time points (Table 1, Figure 2). Second tier contains 23 genes, which responded to IRI in 3 consequent time points (1-36 h, 4 h-10 days, or 24 h-3 month; Figure 2) and third tier contains 4 candidates, which responded to IRI only at 2 time points.

**Figure 3:**
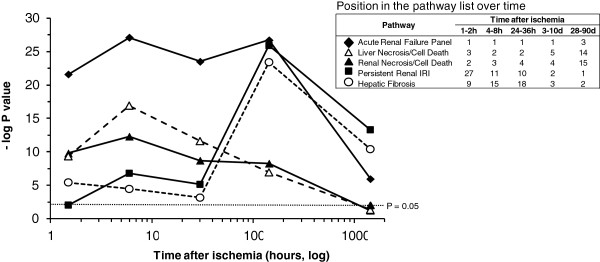
**Ischemia time related pathway analysis of universal interspecies IRI genes.** Genes that were significantly affected by IRI in more than one species at each selected time-point were analyzed by Ingenuity pathway tool. The top three significant pathways from each time-point were identified, and those that were present among top pathways at least twice were plotted against time-course of the IRI. The *y* axis represents negative log of p-value assigned by Ingenuity algorithm (Fisher Exact test) to a pathway. The p-value that corresponds to numeric 0.05 is depicted by the dashed line. The *x* axis represents time course in log_10_ format. The position of each depicted pathway in the list of pathways is shown in the insert table.

To evaluate, whether our tight filtering affected biological relevance of retained 47 gene candidates, we conducted Ingenuity Pathway Analysis (IPA). The IPA of 47 candidates identified *Acute Renal Failure Panel* as the top IRI affected pathway (P < 0.000001, Additional file 4). The pathway analysis for each time-point was conducted using significant candidates, identified by *W*-scoring: 430, 684, 1069, 369, and 93 genes for 1-2 hours, 4-8 hours, 24-36 hours, 3-10 days; 3-5 month after IRI, respectively. The five sets of pathways were generated. The pathways, which were among the top three pathways in at least two consecutive time points are reported in Figure 3 by their significance: “*Acute Renal Failure Panel”*, *“Liver Necrosis/Cell Death*, *Renal Necrosis/Cell Death”*, *“Persistent Renal IRI”*, and *“Hepatic Fibrosis”*. This result further validated our findings and addressed concern that our algorithm puts a strong filter on candidate gene selection, which might inappropriately restrict the biological relevance of the results.

To identify relevance of our candidates to kidney IRI we linked 47 genes to 3 terms: “kidney IRI”, “kidney transplantation”, and “kidney injury” using PubMatrix tool [3]. This approach identified 26 genes (55.3%) as well established kidney IRI genes (≥5 combined citations (CC) of 3 search terms), 10 (21.2%) novel genes (5 > CC > 0) with top candidates *ADAMTS1*, *ANXA2*, and *TYROBP*; and 11 (23.5%) new genes (CC = 0) with top candidates *ARPC1B*, *EGR2*, and *CLDN4* (Additional file 3). The detailed analysis of 20 tier-one candidates revealed significant association of 70% (14 out of 20 genes) with kidney injury, IRI, or transplantation (Table 1), which once again confirmed biological feasibility of our method.

The comparison of 47 gene candidates that were selected by both eGWAS and *W*-scoring algorithms with 28 candidates selected by eGWAS but not *W*-scoring algorithms (Additional file 5) demonstrated that our algorithms filtered out genes with higher bias of rodent models and lower contribution of human models (39.3% of filtered out by our algorithm genes were selected by eGWAS solely based on the rodent models and only 35.7% were selected using human data). On the other hand, among the *W*-scoring retained candidates only 14.9% of genes was selected based on rodent models and more than half of the retained candidates (51.5%) were selected using human data (Additional file 5).

These findings suggest that expression patterns of gene candidates selected by the *W*-scoring algorithm are shared at higher degree amongst species, therefore they might have higher rates of success during further validation in different models. Three of these candidates allowed us to generate three workable hypotheses, one of which was confirmed by an additional study.

Genes in which the whole family was involved in kidney IRI became our primary candidates. The first such gene was *ANXA2* from the annexin A family. Its counterpart *ANXA1* is a well known kidney IRI gene [4,5], which conformingly had the highest kidney IRI association P-value (Figure 1). Based on the knowledge that *ANXA1* protects kidney against IRI via its potent neutrophil anti-migratory activity [4], one can predict the same function for the new candidate *ANXA2*. However, the other well studied annexin *ANXA5*, exerts its antiinflammatory effect via a different mechanism: by blocking phosphatidylserine dependent inflammatory signaling [5]. Therefore, we hypothesize that *ANXA2* will alleviate kidney IRI via its antiinflamatory action, but by which mechanism remains to be investigated. Yet, the reported association of *ANXA2* with tubular injury [6] will steer the future studies towards the epithelial component of kidney.

**Figure 4:**
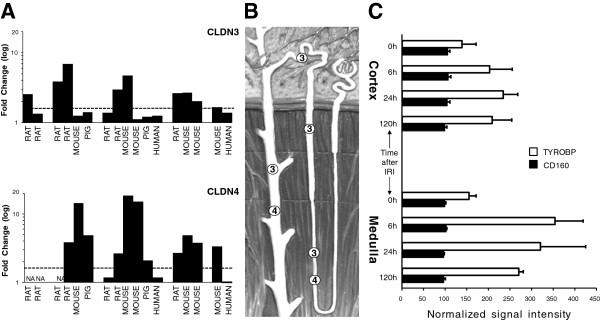
**IRI-induced expression pattern of novel IRI candidate genes *CLDNs *and *TYROBP*. *Panel A***. The expression profiles (*Y axis*) of *CLDN3* and *CLDN4* are presented according to the tested time points (*X axis*). Fold changes (black bars) for each species (labeled on the bottom) are expressed in log_10_ values. The location of each claudin in the distal nephron is depicted in ***Panel B*** (each number corresponds to claudin nomenclature). ***Panel B****.* Diagram of nephron. ***Panel C****.* The pixilated expression values of *TYROBP* and *CD160* genes in cortex and medulla of kidney is represented by horizontal bars. The error bars are standard deviations among 3 samples.

Our next candidate *CLDN4* was from the claudin family. This family has the same basic function of maintaining epithelial barrier integrity and controlling paracellular transport via regulation of tight junctions between neighboring cells. Claudins are highly conserved throughout evolution and are located in close proximity in the human genome [7]. Our candidate *CLDN4* is located within 50 kb of *CLDN3* on chromosome 7. Furthermore, the *CLDN3/CLDN4* tandem demonstrates a coordinate expression in several normal and neoplastic tissues [8]. This was concordant with our data (Figure 4A). Although *CLDN3* did not satisfy Bonferroni threshold, it was ranked by our *W*-scoring algorithm higher (rank 22) than *CLDN4* (rank 78) (Additional file 2). Moreover, the overall trend in its expression pattern was close to that of *CLDN4* (Figure 4A). The slight differences in expression of the tandem *CLDN3*/*CLDN4* members might be attributed to the additional cortex location of *CLDN3* where expression pattern of *CLDN3* might differ (Figure 4B).

Based on the report that the renal protective agent lypoxin, which is thought to exert its protective effects via significant downregulation of numerous IRI-inducible genes, including claudins [9], we hypothesized that the suppression of claudins, which delays the restoration of epithelial integrity, might be beneficial during IRI. We propose a mechanism where the leaky intercellular connections permits nonspecific paracellular dumping of excess inflammatory cytokines into the tubular urinary flow, thus clearing kidney tissue from injurious molecules and decreasing inflammation.

The last candidate, *TYROBP*, had the highest *W*-score among novel/new candidates and satisfied Bonferroni threshold (Table 1 and Figure 1). This gene was also the only new candidate expressed at all time points (Figure 2). *TYROBP* is expressed in circulating immune cells and plays a crucial role in natural killer (NK) cell signaling. To date the known tissue specificity of *TYROBP* is restricted to the immune system and this gene was indirectly linked to kidney IRI via studies of non-specific response by circulating immune cells [10]. There are no reports on the expression of *TYROBP* in kidney tissues (http://www.genecards.org). However, our observations suggested otherwise. Analyzing IRI effects in cortex and medulla of rat kidney reported by Krishnamoorthy et al. [11] we noticed an unexpected pattern in expression of TYROBP. Knowing that >90% of kidney blood flow occurs in the cortex; we anticipated that infiltration of immune cells will occur mainly in this compartment of the kidney, therefore, the expression of *TYROBP* will be higher in the cortex. Surprisingly, we observed the opposite (Figure 4C). Moreover, the marker of NK, *CD160*[12] was not changed in either the cortex or in the medulla, suggesting that the *TYROBP* signal is coming not from immune cells, but rather from kidney tissues. This stipulation was indirectly supported by the fact that expression of *TYROBP* was detected in liver tissue as well [13], an important link, since the transcriptional response of liver to IRI in our study was similar to that of kidney (Figure 3).

**Figure 5:**
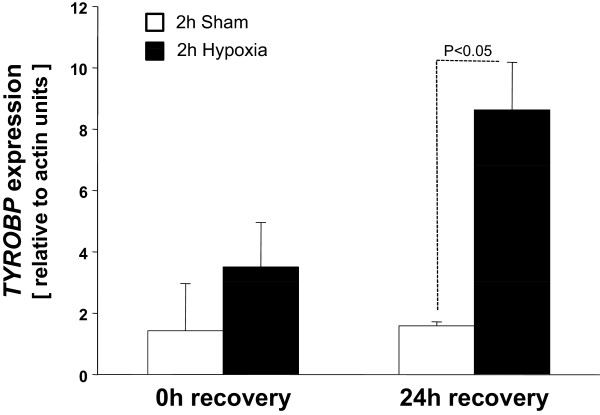
**Expression of TYROBP genes in hypoxic human kidney (HK-2) cells identified by time PCR.** The relative message abundance of TYROBP was detected by real time RT-PCR. The total RNA was obtained from HK-2 cells collected at 0 or 24 hours after hypoxia. The gene expression values are represented by relative to actin-β expression units (mean _(2 independent experiments)_ ± SD). Changes in the gene expression between sham treated and hypoxia treated cells were evaluated by unpaired t-test and P < 0.05 was considered significant.

To test this hypothesis we conducted real time PCR studies in the human kidney cell line HK-2. We simulated IRI in this cell using oxygen-glucose deprivation followed by recovery in regular media. This study demonstrated detectable expression of *TYROBP* in human kidney cells (Additional file 6: Figure S1) and its responsiveness to simulated IRI (Figure 5).

Thus, *TYROBP* is a fascinating therapeutic target, the inhibition of which might protect the kidney from NKs.

## Conclusions

In the presented study, we investigated the response of four different biological systems to kidney ischemia using new pipeline eGWAS — *W*-scoring. This algorithm allowed us to identify reliable IRI candidate genes and select new promising candidates for further studies.

Based on our findings we were able to propose three new hypotheses and provide supporting evidence for one of them.

In summary, our first in the field kidney IRI meta-analysis of 150 microarray samples using eGWAS in combination with our new algorithm of correcting species contribution to the IRI molecular signal, identified 10 novel and 11 new gene candidates, of which 3 described above can lead to discovery of new unsuspected mechanisms of IRI. Moreover, a decreased bias of these genes for the most popular kidney IRI researched rodent model will facilitate investigation of their functions in non-rodent models and accelerate translation of their potential diagnostic and/or therapeutic properties to the bedside.

## Methods

### Data

The kidney IRI datasets were uploaded from the public genomics data repositories Gene Expression Omnibus (GEO, NCBI, (http://www.ncbi.nlm.nih.gov/geo) and ArrayExpress (EBI, http://www.ebi.ac.uk/arrayexpress). The term “kidney ischemia” was submitted to the database and 136 entries were returned (as of April 2012), 24 of which were data series and the remaining data were either array platforms or individual samples.

The inclusion criteria for retrieved data were: 1) the array samples must represent genome wide studies of the whole kidney by established microarray platforms with the number of interrogated sequences >5,000, thus excluding pathway oriented and small custom platforms, which will introduce tissue or biological process bias; 2) the experimental settings must have untreated (placebo or sham) and unmodified (wild type) subjects; 3) the IRI model must be based on the bilateral kidney injury (*i.e.* bilateral clamping), the unilateral models were excluded; and 4) n ≥ 5 per each comparison group (sham + IRI) group.

The studies that satisfied our inclusion criteria are listed in Table 2. Six data series with assigned GEO ID included 9 independent time points of IRI. The specific GEO sample IDs (GSM), which were used for this study and a brief description of experimental settings is listed below starting with the author.

**Table 2:** Summary of microarray data used for meta-analysis

**Authors**	**GEO series**	**Species**	**Effects of ischemia reperfusion injury**				
			**Immediate**	**Early**	**Intermediate**	**Prolonged**	**Distant**
Kusaka *et al.*[14]	GSE5104	Rat	1 h				
Yuen *et al.*[15]	GSE3219	Rat	2 h	8 h			
Hanto *et al.*[16]	GSE14373	Pig		4 h	24 h		
Grigoryev *et al.*[19]	NR*	Mouse		6 h	36 h		
Krishnamoorthy *et al.*[11]	GSE27274	Rat Crtx		6 h	24 h	5d	
		Rat Mdl		6 h	24 h	5d	
Viñas *et al*. [17]	GSE9943	Rat BN			24 h		
		Rat SD			24 h		
Grigoryev *et al.*[21,22]	NR	Human			24 h		
Liu *et al.*[20]	NR	Mouse			24 h		
Ko *et al.*[23]	Pending	Mouse				3d, 10d	28d
Naesens *et al.*[18]	GSE11166	Human					90d

* - not reported to GEO, *Crtx* - cortex, *Mdl* - medulla, *BN* - Brown Norway strain, *SD* - Sprague-Dawley strain.

Kusaka *et al*. [14]: (Rat whole kidney isografts from brain dead donors, shams n = 3; ischemia 6 hours and reperfusion 1 hour n = 3) GSM115035, GSM115037, GSM115038.

Yuen *et al*. [15]: (Rat whole kidney, both renal pedicles were clamped for 40 min, reperfused for 2 (n = 4) or 8 (n = 3) hours with 3 common shams) GSM71831, GSM71835, GSM72108, GSM72109, GSM72114, GSM72117, GSM72229, GSM72253, GSM72281, GSM72502.

Hanto *et al*. [16]: (Pig whole kidney, renal pedicles were clamped for 1 hour and reperfused for 4 (n = 4) or 24 (n = 4) hours with 2 common controls) GSM359391, GSM359392, GSM359393, GSM359395, GSM359397, GSM359399, GSM359401, GSM359403, GSM359405, GSM359407.

Krishnamoorthy *et al*. [11] (Rat cortex and medulla from ischemic (bilateral 20 min clamping) kidneys, which were reperfused for 6 hours (n = 6) 24 hours (n = 6) and 120 hours (n = 6) with 6 common shams. The microarray data was generated for cortex and medula: GSM674258-81.

Viñas *et al*. [17]: (Rat whole kidney from Sprague-Dawley and Brown Norway strains, bilateral clamping for 45 min, reperfusion for 24 hours (n = 3) with 3 shams) GSM251560-61, GSM251586, GSM251588, GSM251591, GSM251593-95, GSM280410-11, GSM280416-17.

Naesens [18]: (Human kidney biopsies before (n = 14) and 90 days after (n = 14) transplantation) GSM281084, GSM281259, GSM281276-77, GSM281346-47, GSM281351-52, GSM281667-73.

The other 4 gene expression profiles were generated, studied and reported by our group [19-23]. Grigoryev *et al*: Mouse whole kidney, bilateral clamping for 1 hour, reperfusion for 6 (n = 3) or 36 (n = 3) hours with corresponding shams (n = 5, n = 3, respectively) [19].

Lui *et al*: Mouse whole kidney, bilateral clamping for 30 min, reperfusion for 24 hours (n = 3) with sham operated controls (n = 3) [20].

Grigoryev *et al*: Human kidney biopsies, 11 paired samples before and 24 hours after transplantation [21,22].

Ko *et al*: Mouse whole kidney, bilateral clamping for 30 min, reperfusion for 3, 10, or 28 days (n = 3 for each time point) with corresponding sham operated controls (total n = 9) [23].

### Standardizing platforms

All single channel arrays were reanalyzed using Significance Analysis of Microarrays (SAM 2.20) [24], which was conducted using 300 permutations without application of arbitrary restrictions [25], as described previously [26]. The fold change values from the multiple channel platform (GPL890) were used directly and log2 ratio p-values were used in place of d score where p < 0.01 was considered significant. The numeric q-values on other platforms were converted to log2 values to facilitate computing of average cross-platform q-value for a given gene. The log incompatible q = 0 we converted to the closest power fraction that SAM called above 0. For example, the lowest fractional call for q in rat dataset from Vinas *et al.* was q = 0.80 and all other genes with higher significance had q = 0. We converted these 0 q-values to 0.1. The lowest fractional call for q-value in rat dataset from Krishamoorthy *et al.* was q = 0.02 and all other genes with higher significance had a q-value = 0. We converted these 0 q-values to 0.01.

The resulting data sets comprised 5 columns: combined probe IDs, gene name, gene symbol, d score, and fold change. The probe IDs across different microarray platforms for mouse, rat, and human were linked using array information library universal navigator (AILUN) tool (http://ailun.stanford.edu) [27], the probes that remained unmatched by AILUN and probes from pig arrays were linked to the AILUN created mouse-rat-human dataset via their gene symbol entries.

### Expression-based genome-wide association study (eGWAS)

The eGWAS was conducted as described previously [28]. Briefly, to estimate differences between groups of samples from IRI subjects and sham controls, the d score, which denotes the standardized change in gene expression [28] was used (calculation of d-score was performed using SAM 2.20 as described above). Genes with an absolute value of d score ≥2 or a fold change ≥2 between shams and IRI were considered significantly dysregulated. There were total of 17,575 known genes across all microarray platforms. For every one of the 17,575 genes, the observed number of microarray experiments in which each gene was significantly dysregulated was derived. Then the P values from the number of positive/negative experiments for each gene and sum of the number of positive/negative experiments for all other genes was calculated using a 2 × 2 chi-square analysis or a Fisher’s exact test as an alternative method, and ranked all of the genes according to their P value (–log_10_P).

### Weighted scoring algorithm

Given that available data was skewed towards rodent models: 13 arrays for mouse and rat models and 4 arrays for pig and human models, there was a need for species bias correction. The straightforward approach of analyzing each species individually and then combining data will greatly decrease statistical power of meta-analysis. Therefore, we completed the meta-analysis of all arrays together and then cross-referenced obtained results with the unbiased gene list, which was generated using a weighted scoring algorithm modified from our previously reported approach [29]. Given that FC is heavily dependent on the platform (*i.e.* data submitted as RMA output provides relatively low FC values even for the most affected genes comparing to GCOS output of the same data) our algorithm favors the unidirectional gene expression over the magnitude of FC and uses the former as a multiplying factor and the later as a summation factor during score calculation. First we averaged fold change and q values for the genes that had multiple probe sets on the same array, then the genes were scored based on the FC values in the log2 increments. The upregulated genes with 1.2 ≤ FC < 2 had score 1; 2 ≤ FC < 4 had score 2; 4 ≤ FC < 8 had score 3; 8 ≤ FC < 16 had score 4; FC > 16 had score 5. The same method was used for the downregulated genes, where scores ranged from –1 to -5.

The scores from different species were converted into the common weighted scores (*W*-score) using our heuristic scoring algorithm [29]. The calculation was based on the score of individual expression signal (*m*) and the number of tested species (*i*) where this signal was detected. The *W*-score (*W*) of a gene was then defined as a sum of *m* reduced (penalized) by the unidirectional coefficient (bracketed expression of the formula). The unidirectional coefficient was computed as a total number of tested species reduced by the number of species, in which a given gene was not changed (*i*_*ns*_) and by the double number of species, in which a given gene was oppositely expressed; then divided by the number of tested species. Thus, if all species demonstrate unidirectional expression for a given gene, the *W* of the gene is simply the sum of individual scores for each tested species.

$$W=\underset{i\geq {2i}_{\text{op}}}{\sum_{i=1}^{m}}m_{i}^{x}\left(\frac{i-\left(i_{\mathrm{ns}}+2i_{\mathrm{op}}\right)}{i}\right)$$

To eliminate the species bias all arrays from the same species were combined, scored using the described algorithm and converted back to the representative FC using the resulting score divided by the number of biological replicates. As an example we offer the description of scoring for the CCL2 gene at time point 24 h - 36 h: FC_rat1_ = 2.0, FC_rat2_ = 2.1 gave (2 + 2)*1 → score = 4 = > 4/2 = 2 or FC = 3.0] [FC_mouse1_ = 1.3, FC_mouse2_ = 7.8 gave (1 + 3)*1 → score = 4 = > 4/2 = 2 or FC = 3.0. The scores of all four species were summed up and multiplied by the number of species: FC_human_ = 2.8, FC_pig_ = 21.4, FC_rat_ = 3.0, FC_mouse_ = 3.0 [score = (2 + 5 + 2 + 2)*4] =44.

The reducing (penalizing) system for the *W* was based on the biological meaningfulness. For instance, the gene *A* which was present on arrays for all four species but was upregulated in three of them and in the fourth species had no change in expression should be scored lower than gene *B* which was upregulated in all four species, but scored higher than gene *C*, which was upregulated in three species but downregulated in the fourth species.

Assuming that all four species from the example above have FC = 2, then we will have the following three scores: [2,2,2,2] (2 + 2 + 2 + 2)×4/4 → score = 8; [2, 2, 2, 0] (2 + 2 + 2 + 0)×(4–1)/4 → score = 4.5; [2, 2, 2, -2] (2 + 2 + 2–2)×(4–2)/4 → score = 2. The detailed example of scoring matrix is provided in Additional file 7.

For candidate gene selection throughout all time points the universal cutoff for the score value was set at 2, which represents 2 ≤ FC < 4; and false discovery rate (q-value) was set at the level of 10%. The resulting gene list was filtered using *W* > 10 (which represents FC = 2 in all five time-points) and q < 10.

### Pathway analysis

The pathway analysis, which identifies the most relevant biological processes to a specific list of candidate genes, was conducted using the Ingenuity Pathways Knowledge Base tool (IPA, Ingenuity Systems, Inc., Redwood City, CA.) as described previously [19,30]. For this analysis we generated individual gene lists for each time point, therefore the cutoffs for the score value were also set individually and were equal to the number of tested species in a particular time point (Additional file 4). Gene symbols from the resulting gene lists were submitted to the IPA and analyzed using “Tox Lists” tool. Significance of the identified pathways was tested by the IPA imbedded Fisher Exact test p-value [31].

### Automated literature search

PubMatrix (multiplex literature mining tool) analysis [3] was conducted as described previously [32]. We restricted our search to human symbols approved by HUGO Gene Nomenclature Committee (HGNC), which were enriched by all aliases and former (discontinued) symbols for selected candidate genes (http://www.genenames.org). The symbols with the hyphen and double names separated by a slash were filtered out. As a result our 47 candidates were combined with 109 aliases and cross-referenced with terms “kidney injury”, “kidney ischemia reperfusion injury”, and “kidney transplantation”. The biomedical literature reference count for a given gene was represented by the highest number among all aliases of a given gene. The genes with number of references N ≥ 5 were considered to have established association with kidney IRI, 5 > N > 0 were considered novel, and N = 0 were considered new.

### Evaluating the effects of the weighted scoring algorithm

To evaluate the effect of our new algorithm on the selection of gene candidates, we compared the overall species signal contribution to the selected gene candidates with candidates that were filtered out by our method. The fraction of rodent model contribution and the fraction of human model contribution were used as the end-points for comparison.

### Oxygen-Glucose deprivation of human kidney (HK-2) cells

Human, kidney proximal tubule (HK-2) cells were purchased from American Type Culture Collection (ATCC®, cat# CRL-2190, LOT: 59538658) and grown on T75 flask until confluent.

The hypoxia experiment was conducted as described previously [33]. Briefly, cells were washed with warmed PBS and OGD solutions, and covered with oxygen deprived OGD solution. The airspace in the flask was flashed with nitrogen to remove remaining oxygen. The flack was capped and placed into HeraCell incubator at 1% O_2_ and 37°C. After 2 h flasks were removed and OGD solution was either replaced with growing media (24 h recovery) or treated with lysis buffer from mir-Vana kit (0 h recovery) for RNA extraction.

### Real-time RT-PCR

Transcript levels of TYROBP in HK-2 cells from 2 independent experiments were measured as previously described with slight modification according to new manufacturer protocols [29]. Briefly, the 384-well microtiter plate setting of a ViiA™ 7 Real-Time PCR System (Applied Biosystems) was employed. TaqMan® Predeveloped Assay Reagent human β-actin (REF 4326315E, probe dye VIC-MGB) was used as an internal control for normalization. TaqMan® Gene Expression assay for human *TYROBP* was purchased from Applied Biosystems Inc. (Hs00182426_m1). All experimental protocols were based on manufacturer’s recommendation using the TaqMan® Universal Master Mix II (P/N 4440039). A relative quantitative method was used to calculate *TYROBP* transcript levels relative to the actin expression. The significance of the obtained difference in signal between sham and hypoxia treated cells was assessed using t-test where p < 0.05 was considered significant.

## Competing interests

The authors declare that they have no competing interests.

## Authors’ contributions

DNG, HR and SQY conceived of the study. LQZ and DPH participated in its design. DRC carried out mouse and tissue culture studies. PH carried out real time PCR studies. DNG and SQY did statistical analyses of all data and drafted the manuscript. All authors read and approved the final manuscript.

## Pre-publication history

The pre-publication history for this paper can be accessed here:

http://www.biomedcentral.com/1471-2369/14/231/prepub

## Supplementary Material

Additional file 1 The working examples of new scoring algorithm. Click here for file

Additional file 2 List of 75 gene candidates identified by chi-square test. Click here for file

Additional file 3 List of 116 gene candidates identified by new scoring algorithm. Click here for file

Additional file 4 List of 47 gene candidates identified by both chi-square test and new scoring algorithm. Click here for file

Additional file 5 List of interactive networks identified by the Ingenuity software. Click here for file

Additional file 6: Figure S1 Species correction using new algorithm. Click here for file

Additional file 7 Amplification plots of real time PCR studies. Click here for file
