# Supplementary material for: Meta-analysis of molecular response of kidney to ischemia reperfusion injury for the identification of new candidate genes
Source: BMC Nephrol. 2013 Oct 24;14:231. doi: 10.1186/1471-2369-14-231 (PMC4016589; doi:10.1186/1471-2369-14-231)
Supplement: Additional file 6: Figure S1 — Species correction using new algorithm. [file 1471-2369-14-231-S6.pdf]

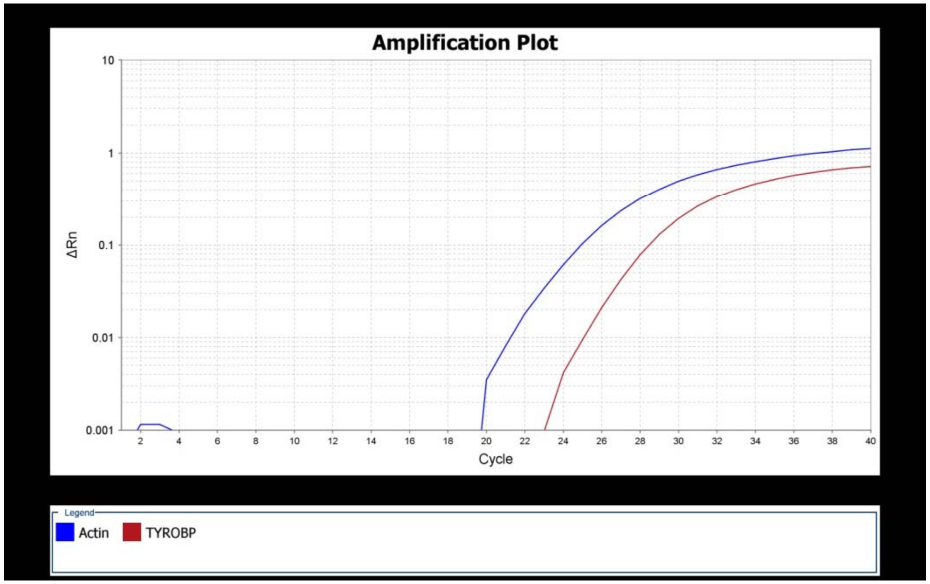


**2 h normoxia**

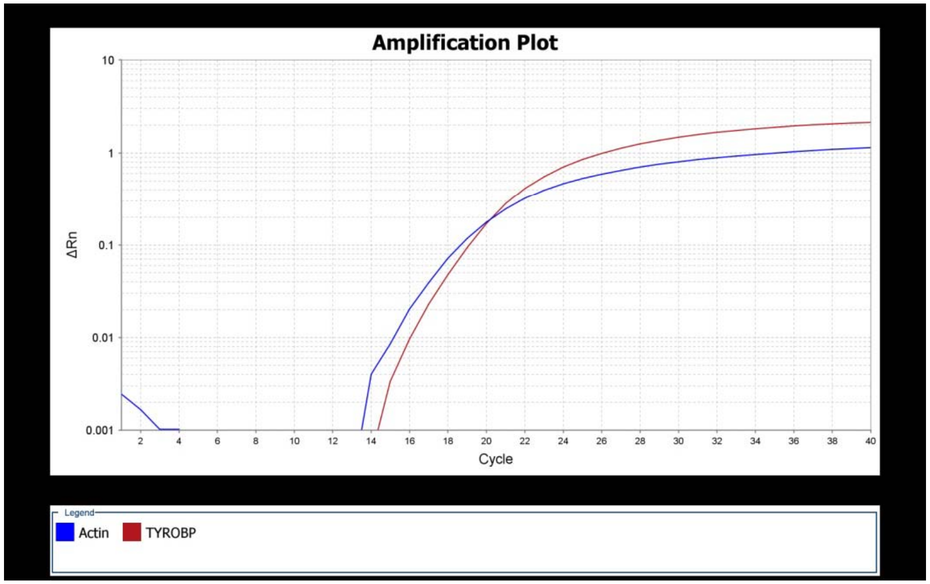


**2 h hypoxia**

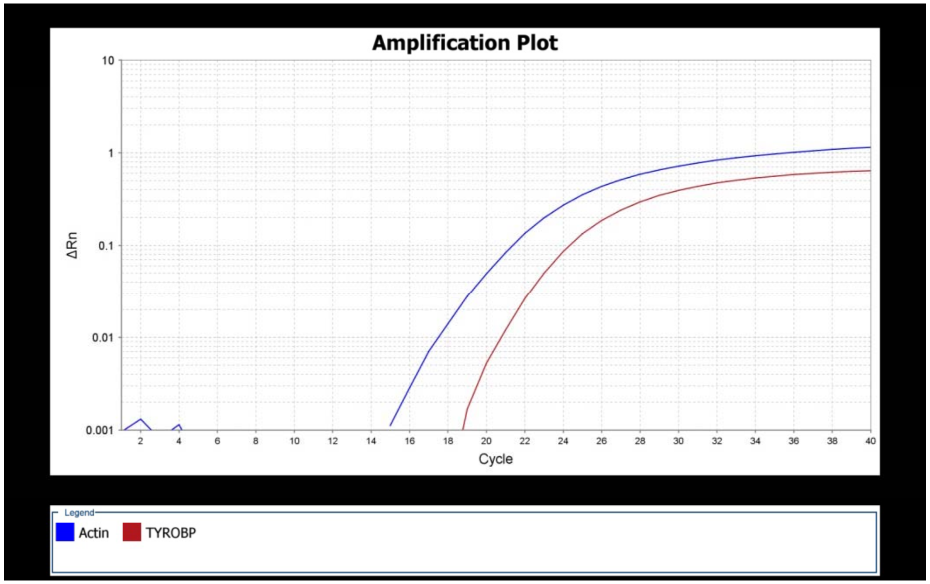


**2 h normoxia +**
**24 h recovery**

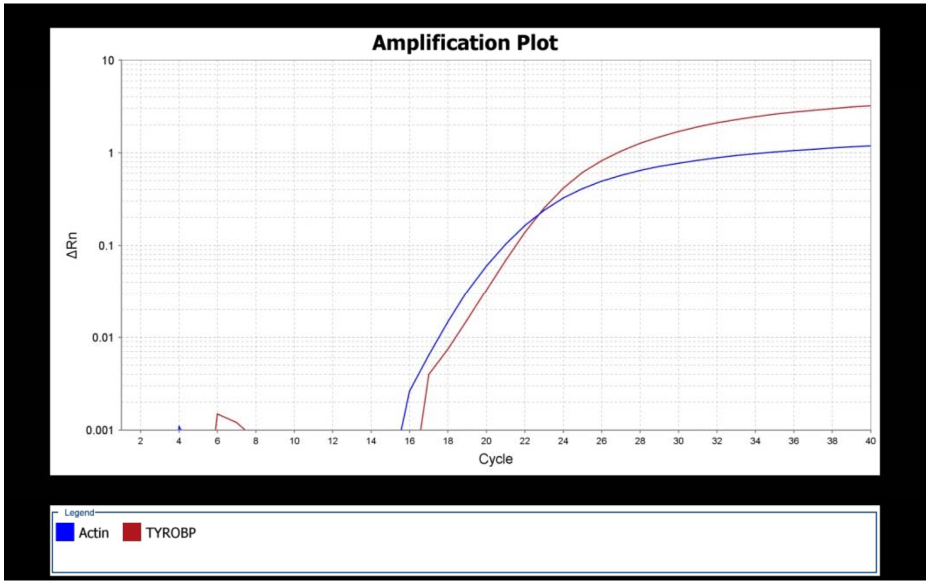


**2 h hypoxia +**
**24 h recovery**
